# Sex-Related Differences in Circular RNA Expression in Multiple Sclerosis: A Pilot Study

**DOI:** 10.1007/s12031-026-02548-3

**Published:** 2026-05-26

**Authors:** Valeria Lodde, Ignazio Roberto Zarbo, Gabriele Farina, Enrico Zoroddu, Paolo Solla, Giuseppe Delogu, Myriam Gorospe, Matteo Floris, Ilaria Campesi, Maria Laura Idda

**Affiliations:** 1 Department of Biomedical Sciences, University of Sassari, Sassari, 07100 Italy; 2 Department of Medicine, Surgery and Pharmacy, University of Sassari, Sassari, 07100 Italy; 3 Unit of Clinical Neurology, AOU Sassari, Sassari, 07100 Italy; 4 Laboratory of Genetics and Genomics, National Institute on Aging Intramural Research Program, National Institutes of Health, Baltimore, MD 21224 USA; 5 Department of Medical Sciences and Public Health, University of Cagliari, Cagliari, 09042 Italy

**Keywords:** Circular RNAs, Multiple sclerosis, Sex differences, Gene regulation, Immune regulation

## Abstract

Multiple Sclerosis (MS) is an inflammatory autoimmune disease of the central nervous system (CNS), with a female-to-male ratio of up to 3:1. Sex differences in immune responses and neurodegeneration influence disease susceptibility and progression. Circular RNAs (circRNAs) are emerging regulators of CNS and immune cell functions; however, the mechanisms underlying their regulation and dysregulation in MS remain poorly understood. Given the relevant role of sex-related differences in MS, in this pilot study we re-analyzed a previously published dataset by performing a sex-stratified analysis. Specifically, we focused on identifying circRNAs that are differentially expressed in female MS patients compared to male MS patients. We identified 33 differentially expressed circRNAs in peripheral blood mononuclear cells: 27 were more expressed and 6 were less expressed in female MS patients as compared to male MS patients. The discovery dataset was validated in an independent cohort of 8 female and 8 male relapsing-remitting (RR) MS patients confirming sex-dependent differences in the expression of five circRNAs. Among these, *hsa_circ_0140253* was significantly elevated, while *hsa_circ_0029426*,* hsa_circ_0005354*, and *hsa_circ_0002082* were significantly reduced. Notably, *hsa_circ_0140253* expression was markedly increased in patients with a higher level of disability and in progressive forms of MS, and across various disease-modifying therapies used in MS treatment. This exploratory study provides preliminary evidence of sex-related differences in circRNA expression in MS and their association with disease severity and DMT exposure. Our findings suggest that circRNA expression is influenced by multiple factors, underscoring the importance of considering sex and clinical variables in future studies. Further validation in larger cohorts is needed to confirm these observations and clarify the role of circRNAs in MS pathogenesis.

**Graphical Abstract:**

**Figure:**
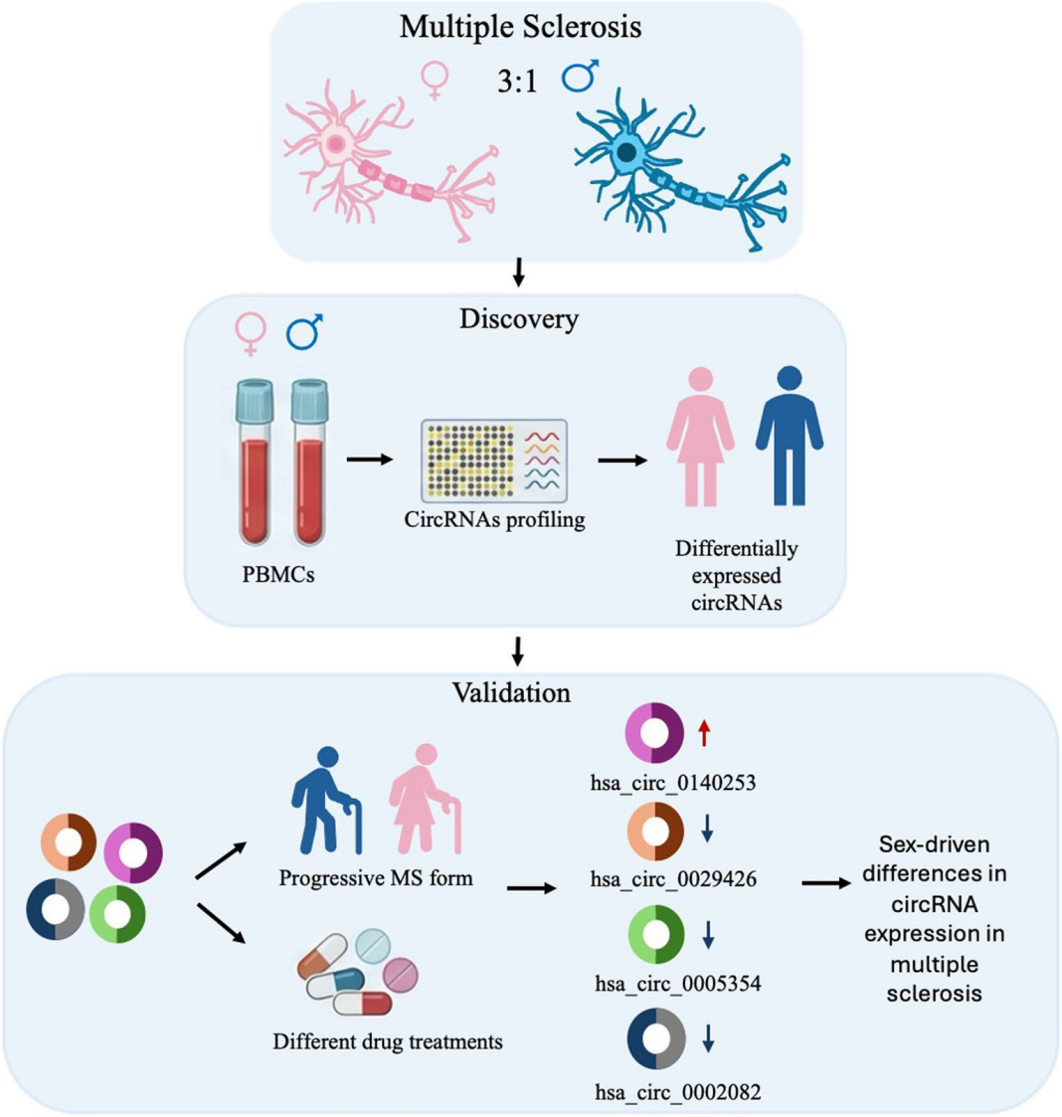
A sex-stratified analysis of PBMC samples from multiple sclerosis (MS) patients identifies distinct circRNA expression patterns between females and males. Sex-related circRNA differences are associated with disease severity and exposure to disease-modifying therapies. We identified 33 circRNAs differentially expressed between female and male MS patients, highlighting the increased levels in female of hsa_circ_0140253 and the reduction of hsa_circ_0029426, hsa_circ_0005354, and hsa_circ_0002082. These preliminary findings support the need for larger, sex-stratified and functional studies to better define the contribution of circRNAs to MS pathogenesis.

**Supplementary Information:**

The online version contains supplementary material available at 10.1007/s12031-026-02548-3.

## Introduction

Multiple sclerosis (MS) is a chronic autoimmune and demyelinating disease of the central nervous system (CNS) affecting 3 million persons worldwide (Reich et al. 2018). MS is driven by an autoimmune inflammatory response which leads to axon demyelination and loss, plaque formation, and neurodegeneration (Schaeffer et al. 2015). Clinically, MS can be classified into four subtypes: clinically isolated syndrome (CIS), relapsing-remitting MS (RRMS), primary progressive MS (PPMS), and secondary progressive MS (SPMS) (Sand 2015).

RRMS, which accounts for approximately 80% of cases, is primarily defined by episodic exacerbations succeeded by intervals of partial or full neurological remission. RRMS can progress to SPMS (Sand 2015). Less frequently (∼10%), patients experience a progressive form of MS, PPMS, in which continuous progression of disability occurs since onset (Brown et al. 2019). Although clinically similar to SPMS, PPMS is characterized by a later age of onset and is associated with fewer white matter (WM) lesions. Additionally, PPMS tends to show reduced inflammatory activity and greater myelin and axonal damage compared to SPMS (Miller and Leary 2007).

It is now widely accepted by the scientific community that genetic background, sex, and environmental factors (e.g. Epstein-Barr virus, vitamin D deficiency and smoking) can contribute to MS pathogenesis (Frau et al. 2021; Steri et al. 2017; Urru et al. 2020). Indeed, genome-wide association studies (GWAS) have identified HLA-DRB1*15:01 as a key genetic factor contributing to MS susceptibility; moreover, over 230 HLA-independent variants have been associated with MS risk suggesting a relevant role of a plethora of loci mainly implicated in immunological and neurological pathways (International Multiple Sclerosis Genetics Consortium 2019; Olsson et al. 2016). Moreover, it is now widely recognized that sex represents a fundamental determinant of health, shaping disease susceptibility, progression, and clinical manifestations across many complex disorders, including MS (Feng et al. 2024; Gilli et al. 2020).

MS affects women more often than men, by a ratio of approximately 3:1 (Whitacre et al. 1999); this observation is in line with data supporting the increased susceptibility of women to several autoimmune diseases and suggests a fundamental sex-dependent mechanism driving disease onset (Bove and Chitnis 2013). Of note, RRMS has a strong female prevalence, while males who develop MS are more susceptible to neurodegeneration and progressive forms of the disease (Alvarez-Sanchez and Dunn 2023). Sex differences in MS are attributable to differential gene expression related to the presence of sex chromosomes as well as to the different production of sex hormones (Voskuhl and Gold 2012). The effects of sex hormones on immune responses in MS and in experimental autoimmune encephalomyelitis (EAE), the most commonly used animal model of MS, have been widely documented in the literature (Voskuhl 2020; Voskuhl et al. 2020; Wisdom et al. 2013). Interestingly, the effect of sex chromosomes on immune responses has also been observed in MS models, with the female genotype (XX) conferring a more pro-inflammatory response than the male genotype (XY) (Borziak and Finkelstein 2022). The increasing female-to-male ratio in MS in recent decades suggests changes in gene–environment interactions (Voskuhl and Gold 2012). Supporting X-chromosome involvement, dysregulation of X-chromosome inactivation (Xi) predisposes women to MS (Knudsen et al. 2007), and 15–23% of X-linked genes escape Xi (Libert et al. 2010).

Several autoimmune susceptibility genes are located on the X chromosome, such as TLR7, TLR8, IL2Rγ, FOXP3, CXCR3, and CD40L (Greer and McCombe 2011). TLR7 and TLR8, which escape X-inactivation in immune cells, contribute to MS pathogenesis by altering IFN-α promoting axonal damage through increased neutrophil and leukocyte infiltration in MS animal models (Hayashi et al. 2012; Mycko et al. 2014; Souyris et al. 2018; Youness et al. 2023). IL2Rγ is upregulated in the MS brain, while FOXP3, a key regulator of Treg cells, is reduced in MS (Huan et al. 2005; Peerlings et al. 2021). CXCR3 supports T-cell accumulation in the CNS, and the CD40–CD40L pathway plays a central role in modulating immune responses and is associated with MS progression (Sorensen et al. 2002; Vermersch et al. 2024). Today the underlying causes of MS remain poorly understood, but a role for non-coding RNA (ncRNA) including circular RNAs (circRNAs), and RNA processing have been recognized among the molecular mechanisms involved in MS (Mohammed 2020). In this context, one relevant example is the Gasdermin B (GSDMB) locus, a gene previously associated with asthma susceptibility and autoimmune disorders (Cardamone et al. 2017). Alternative splicing of GSDMB gives rise to hsa_circ_0106803, which has been reported to be highly expressed in several brain regions and upregulated in peripheral blood mononuclear cells (PBMCs) from patients with RRMS (Cardamone et al. 2017). In line with a functional involvement of this gene in immune regulation, reduced expression of GSDMB in memory CD4 + T cells has been associated with increased production of pro-inflammatory cytokines, including TNF, IL-13, and IL-16, suggesting a potential contribution of GSDMB-related pathways to MS immunopathogenesis (Schmiedel et al. 2016). Supporting circRNA dysregulation in MS comes from transcriptomic studies in peripheral blood leukocytes, which have reported altered expression of several circRNAs. In particular, *hsa_circ_0005402* and *hsa_circ_0035560*, both originating from the ANXA2 locus, were found to be significantly downregulated in MS patients, further indicating that circRNA-mediated regulatory mechanisms may be involved in disease-related immune processes (Iparraguirre et al. 2017).

CircRNAs can serve as platforms for the binding of microRNAs (miRNAs) and RNA-binding proteins (RBPs), and they can regulate transcription, mRNA metabolism, and translation (Li et al. 2019; Lodde et al. 2023). Importantly, circRNAs expression level can be influenced by disease progression and associated therapies (Lodde et al. 2024; Mohammed et al., 2023). We recently performed an extensive analysis of circRNAs dysregulated in MS patients and reported that *hsa_circ_0018905* was significantly decreased in MS patients compared to healthy control (HC) subjects; furthermore *hsa_circ_0018905* was significantly downregulated in patients with higher level of disability and in SPMS cases (Lodde et al. 2024).

Given the relevant role of sex-related differences in MS, in this pilot study we re-analyzed one of our previously published datasets (Lodde et al. 2024) by performing a sex-stratified analysis. Specifically, we focused on identifying circRNAs that are differentially expressed in female MS patients compared to male MS patients. As secondary objectives, we also evaluated sex-related differences according to disease stage and type of pharmacological treatment.

Among the circRNAs showing sex-specific expression patterns in MS, *hsa_circ_0140253* was found to be more expressed in PBMCs and serum samples from female MS patients compared to male patients, whereas *hsa_circ_0029426* exhibited reduced expression in female MS patients compared to male MS patients.

Interestingly, *hsa_circ_0140253* levels were markedly increased in patients with higher levels of disability, in progressive forms of MS, and across various disease-modifying therapies (DMTs) used in MS treatment. These preliminary findings suggest a possible association between circRNAs and sex-related differences in MS. However, these results require cautious interpretation and further validation. Additional studies are needed to confirm these observations and to better clarify the potential contribution of sex-related differences in circRNA expression to MS pathogenesis.

## Materials and Methods

### Study Design Female MS vs. Males MS

In this pilot study whole-blood and serum samples were collected from all the individuals enrolled in the study. The study was structured to include four distinct cohorts: (i) a discovery cohort consisting of 5 newly diagnosed MS patients (3 females and 2 males), without comorbidities and not undergoing any pharmacological treatment, previously described in Lodde et al. (Lodde et al. 2024); (ii) a validation cohort, comprised of 8 female and 8 male adults diagnosed with MS without comorbidities and not undergoing any treatment; (iii) a third cohort of MS patients with mild and severe disease comprising 8 RRMS patients (4 females and 4 males) newly diagnosed with Expanded Disability Status Scale (EDSS) < 4.5, and 8 SPMS patients (4 females and 4 males) with EDSS > 4.5; (iv) a fourth cohort of MS patients treated with different drugs which includes 8 MS untreated (UNT) patients with EDSS < 4.5 (4 females and 4 males), 8 MS patients (4 females and 4 males) treated with dimethyl fumarate (DMF) and with EDSS < 4.5, 8 MS patients (4 females and 4 males) treated with fingolimod (FTY) and with EDSS < 4.5, 8 MS patients (4 females and 4 males) treated with interferon (IFN) and with EDSS < 4.5, 8 MS patients (4 females and 4 males) treated with natalizumab (NAT) and 8 MS patients (4 females and 4 males) treated with teriflunomide (TER) and with EDSS < 4.5 (Supplemental Figure S1). All MS patients were recruited from the University Hospital of Sassari (Italy). Total RNA, derived from PBMCs, was isolated as described below and used for the initial microarray analysis (Lodde et al. 2024) and validation steps (ii, iii and iv) using reverse transcription (RT) followed by quantitative (q)PCR.

### RNA Extraction

PBMCs have been isolated starting from 10 ml of blood from each donor using Vacutainer CPT tubes (BD), in line with the manufacturer’s protocol. RNeasy Mini Kit (Qiagen) was used to extracted total RNA from the PBMCs. Extracted RNA was dissolved with 50 µl RNase-free water and the concentration was measured with NanoDrop2000 (Thermofisher, Waltham, MA, USA). Human serum samples were collected from patients in the validation set and processed as described below.

Vacutainers with clot activator were kept upright for 60 min to allow red blood cells (RBCs) to form a clot. The RBC clot was then separated by centrifugation at 1,000 × g for 10 min, and the serum was carefully collected. The resulting supernatant was centrifuged at 5,000 × g for 10 min to eliminate cell fragments and other debris. Serum samples were then pooled and stored in 300 µl aliquots at − 80 °C until further analysis. RNA from the serum was isolated using miRNeasy Serum/Plasma Advanced Kit (Qiagen). Extracted RNA was dissolved with 20 µl RNase-free water and the concentration was measured with Nano Drop2000 (Thermofisher, Waltham, MA, USA). All the samples were adjusted to the same concentration, and 50 ng of RNA was used as input for the cDNA synthesis.

### RNA Digestion, Amplification, Labeling, and Hybridization

Total RNA extracted from the discovery cohort was used for microarray analysis, as previously reported (Lodde et al. 2024) (Arraystar Inc.). All samples included in the discovery phase were processed simultaneously following the same experimental workflow and analytical pipeline under standardized conditions to minimize technical variability. RNA quality was evaluated by Arraystar, and all samples passed quality control and proceeded to the subsequent step. In brief, the RNA was treated with RNase R (Epicentre, Inc) to remove linear RNAs. The enriched circRNAs were transcribed into fluorescently cRNA using a random priming approach, followed by amplification using the Arraystar Super RNA Labeling Kit (Arraystar). The labeled cRNA products were purified using the RNeasy Mini Kit (Qiagen).

Labeled cRNA sample (1µg) was fragmented using a Fragmentation Buffer. After 30’ incubation at 60 °C, 25 µl of Hybridization Buffer was added to dilute the labeled cRNA, and 50 µl of the hybridization solution was dispensed into a gasket slide, subsequently assembled onto the circRNA expression microarray slide. The slides were incubated for 17 h at 65 °C in an Agilent Hybridization Oven. After hybridization, arrays were washed, fixed, and scanned using the Agilent Scanner G2505C (Agilent Technologies).

The resulting images were analyzed using Agilent Feature Extraction software (version 11.0.1.1) to obtain raw signal data. Quantile normalization and additional data processing were performed with the R software package (Arraystar). Following normalization, low-intensity filtering was applied. The statistical significance of the differentially expressed circRNAs was estimated by t-test. CircRNAs showing an absolute fold change of ≥ 1.5 and p-values ≤ 0.05 between female and male MS patients were considered significantly differentially expressed. Hierarchical clustering of these circRNAs was performed using Java Treeview (Stanford University School of Medicine).

### Reverse Transcription (RT)-Quantitative (q)PCR Analysis

First-strand cDNA was synthesized using Maxima reverse transcriptase (Thermo Fisher) and random hexamer primers. cDNA was diluted 1:10 and used as a template for RT-qPCR using the SYBR Green master mix (Kapa Biosystems). Relative RNA expression levels were determined using the 2^−ΔΔCt method, and the levels of *GAPDH* mRNA were used for normalization. Data are presented as fold induction (FI) values. The gene-specific primer sequences are provided in Supplemental Table 1.

### Prediction of circRNA-miRNA and circRNA-RBP Interactions

circRNA-associated miRNAs were identified using MiRanda software (v3.3a) (Enright et al. 2003). Potential RBPs that might interact with the validated circRNAs were identified using the Circular RNA Interactome (CircInteractome, https://circinteractome.nia.nih.gov/), based on CLIP data sets (Dudekula et al. 2016).

### CircRNA-microRNA-mRNA Network

miRanda software (v3.3a) was used to identify potential miRNA targets for each differentially expressed circRNA (Enright et al. 2003).We further explored the biological roles of each circRNA by predicting proteins that might be influenced through circRNA-miRNA-mRNA regulatory networks. mirTarBase (release 9.0 beta) were used to identify miRNA-mRNA interactions (Huang et al. 2022). We focused the analysis on mRNA encoding for MS-related genes highlighted by GWAS results obtained by the International Multiple Sclerosis Genetics Consortium (International Multiple Sclerosis Genetics Consortium 2019). Finally, with Cytoscape v3.9.0 the circRNA-miRNA-mRNA networks were visualized (Shannon et al. 1971).

### Replication of *LOC389906* Gene Expression in Public Dataset

Public RNA-Seq datasets were obtained from the Gene Expression Omnibus (GEO) database. We selected two datasets from MS patients (GSE250453 and GSE235357) including data originated from PBMCs of 14 female and 5 male. Only baseline samples from untreated patients were considered for the analysis. To analyze HC we selected the dataset GSE260981 which included RNA samples from PBMCs of 9 female and 12 male HC individuals. Differential gene expression analysis was performed using the DESeq2 (Love et al. 2014) package from the R software (version 1.46.0). Raw read counts were used as input, and normalization was performed using the median-of-ratios method implemented in DESeq2. Genes with an adjusted p-value (padj) ≤ 0.05 were considered significantly differentially expressed.

### Statistics and Data Representation

This was a pilot study conducted on a convenience sample based on the number of available patients who reflected the inherent characteristics of the clinical population under investigation, and was intended to assess the feasibility/acceptability of an approach to be used in a larger scale study.

Data were presented as the means ± standard deviations; GraphPad Prism 9 (GraphPad Software, La Jolla, CA) was used to analyze all experimental data. Relative gene expression levels were calculated using the comparative Ct method. Data were normalized to the reference group (male samples), which was set to 1, and all other groups were expressed as fold changes relative to facilitate comparison across conditions. Differences in RNA levels among groups were evaluated using a two-tailed Student’s *t*-test and *P* ≤ 0.05 was considered statistically significant.

## Results

### Analysis of the MS Patients Cohort

**Fig. 1:**
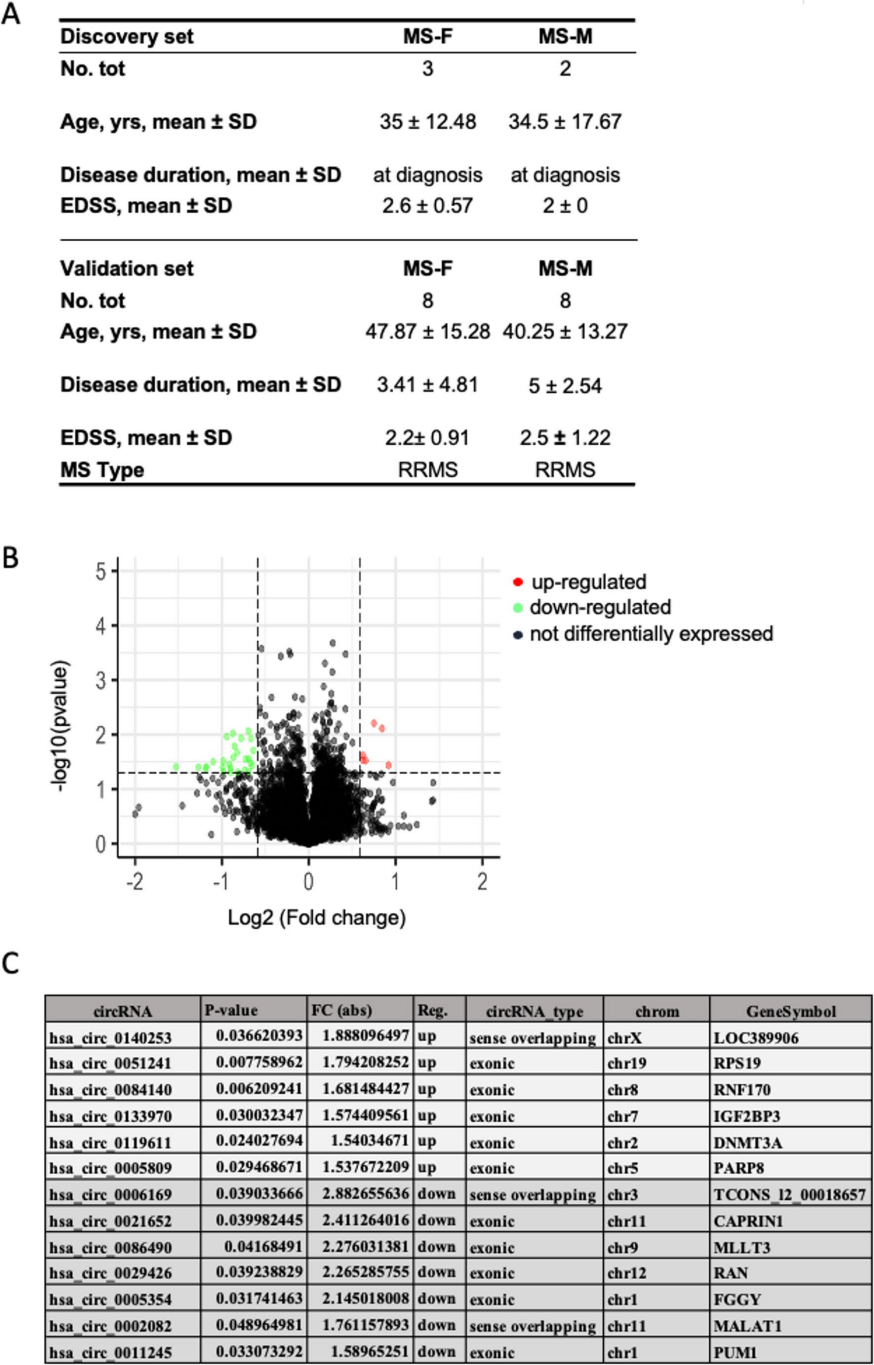
Differentially expressed circRNAs in female versus male MS patients, as identified by circRNA array analysis. (**A**) Main characteristics of the individuals enrolled in the discovery set and the validation set. (**B**) Volcano plots showing circRNAs that are more or less expressed in female MS samples compared to male MS samples. The red (up) and green (down) dots in the plot represent the significative differentially expressed circRNAs. (**C**) Table showing the list of circRNAs differentially expressed, depicting the top 7 elevated and 10 reduced. Abbreviations: MS-F, Multiple Sclerosis Female; MS-M, Multiple Sclerosis Male; SD, standard deviation; EDSS, Expanded Disability Status Scale; RRMS, relapsing–remitting MS.

To preliminary explore whether sex influences the levels of circRNAs in females and males we used a previously published microarray dataset (Lodde et al. 2024) that analyzed circRNAs expression in PBMCs of RRMS patients using the Human Circular RNA Array (Arraystar circRNAs). The RRMS patients enrolled were composed of 3 females and 2 males, age- matched, who comprised the discovery cohort described in Fig. 1A (discovery set). All patients included in the discovery cohort were diagnosed with RRMS, had an EDSS < 2.5 and were enrolled at the time of diagnosis. For the validation step, the cohort was expanded as described in Fig. 1A (validation set), comprising 8 females and 8 males diagnosed with RRMS and with an EDSS of 2.2 ± 0.91 for females and 2.5 ± 1.22 for males. To reduce potential confounding variables during analysis, all donors were negative for comorbidities and had not received any drug treatment. To minimize geographic variability, all the enrolled individuals were from Sardinia, with at least three grandparents of Sardinian ethnicity.

The Arraystar circRNAs platform was designed to identify 13,617 circRNAs, which were analyzed using the Agilent Feature Extraction software as described in Lodde et al. (Lodde et al. 2024). A total of 64 circRNAs were found to be differentially expressed in MS patients at the time of diagnosis, as compared to HCs, of which 53 were downregulated (Lodde et al. 2024). When comparing female and male RRMS patients, the analysis of the dysregulated circRNAs revealed a total of 33 differentially expressed circRNAs, 6 higher and 27 lower in females compared to males (Fig. 1B).

The levels of circRNAs showing the most significant differences between females and males with MS in the discovery cohort are reported in Fig. 1C. *Hsa_circ_0140253* was found to be the most upregulated (FI = 1.88; *p* = 0.036), while *hsa_circ_0006169* was the most downregulated (FI = 2.88; *p* = 0.039). The parental genes from which circRNAs originate are also indicated in Fig. 1C and underline the fact that most circRNAs identified originate from protein coding genes. To ensure that the observed differences are specific to MS patients we analyzed the differentially expressed circRNAs in females compared to males in HCs (**S**upplemental Figure S2). Interestingly, we found no overlap in differentially expressed circRNAs when comparing males and females affected by MS relative to HCs.

**Fig. 2:**
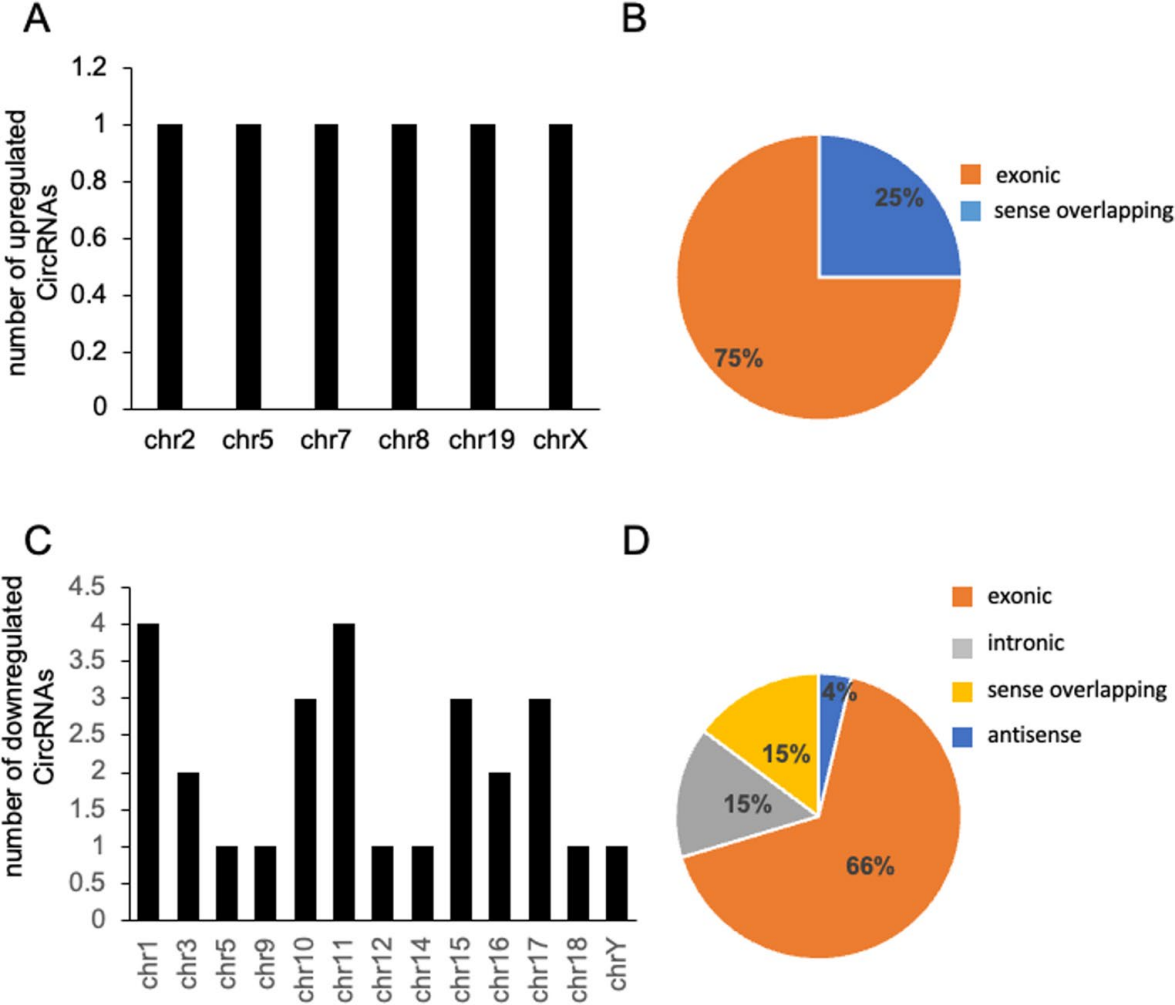
Characteristics of circRNAs identified in PBMCs from female versus male MS patients. (**A**) Distribution of significantly more expressed circRNAs across chromosomal locations. (**B**) Class distribution of upregulated circRNAs based on genomic origin. (**C**) Distribution of significantly reduced circRNAs according to chromosomal location. (**D**) Class distribution of downregulated circRNAs based on genomic origin

Next, we evaluated the distribution of sex-dependent differentially expressed circRNAs across chromosomes and found that they are distributed across all chromosomes, with chromosomes 1 and 11 containing the highest number of differentially expressed circRNAs (8 total). More expressed circRNAs were generated from genes located on chromosomes 2, 5, 7, 8, 19, and X, while less expressed circRNAs were generated from genes located on multiple chromosomes, with chromosomes 1 and 11 containing the highest number (Fig. 2A-C). Most of the identified circRNAs mapped within exons; this group comprised 75% of the elevated circRNAs and 66% of the downregulated circRNAs (Fig. 2B-D).

### RT-qPCR Validation of circRNA Expression Profiles

The validation step for the candidate circRNAs was performed using RT-qPCR with divergent primers (Supplemental Table 1) spanning the circRNA junction. RNA was isolated from PBMCs purified from samples of the validation cohort (Fig. 1A), consisting of 8 females and 8 males affected by RRMS.

**Fig. 3:**
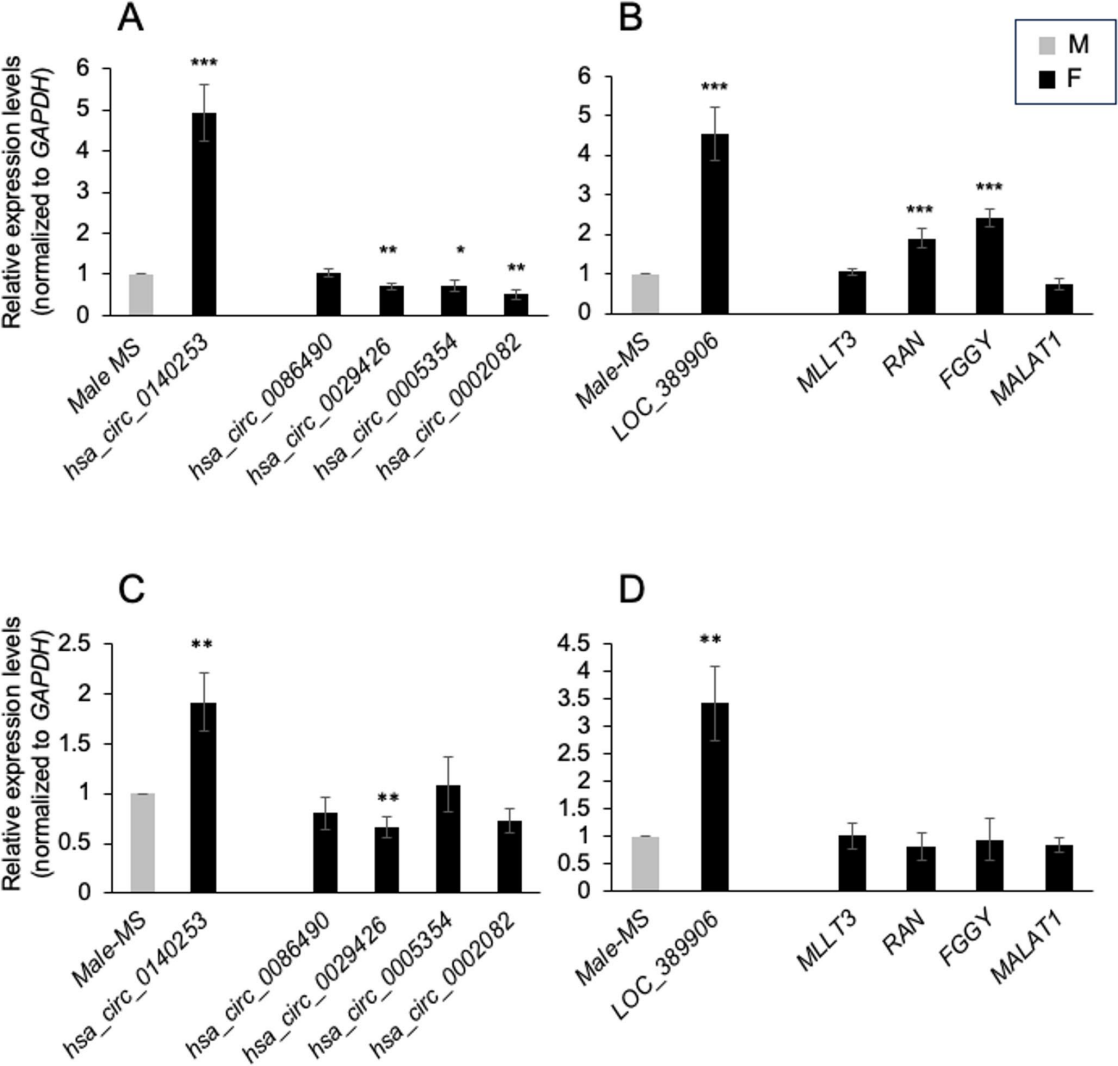
Validation of the circRNAs identified in PBMCs and serum from female versus male MS patients. (**A**) Expression levels in PBMCs of one upregulated and four downregulated circRNAs and (**B**) the corresponding cognate linear mRNAs were measured by RT-qPCR analysis. **(C**,** D)** The levels in serum of one upregulated and four downregulated circRNAs (**C**) and the corresponding cognate linear RNAs (D) were measured by RT-qPCR analysis. The levels of circRNAs and RNAs were normalized to *GAPDH* mRNA levels. Data are the means ± standard deviation (+ SD) from 8 samples for each sex. Statistical significance between groups was assessed using a two-tailed Student’s t-test (* *p* < 0.05, ** *p* < 0.01, *** *p* < 0.001)

The expression trend of the selected circRNAs was consistent with the microarray results. RT-qPCR data revealed that *hsa_circ_0140253* (FI = 4.92; *p* = 7.55 × 10^− 6^), was significantly more expressed in the female group compared to the male group. Moreover, *hsa_circ_0029426* (FI = 0.71; *p* = 0.002), *hsa_circ_0005354* (FI = 0.71; *p* = 0.043), and *hsa_circ_0002082* (FI = 0.52; *p* = 0.001) were significantly less expressed in the validation cohort. No significant difference in the levels of *hsa_circ_0086490* was found in RRMS females compared to RRMS males (Fig. 3A). Next, we examined the relative expression of the host linear transcript for each circRNA. In contrast to the reduction in levels of the cognate circRNAs, the levels of *RAN* and *FGGY* mRNAs were elevated while *MALAT1* did not significantly change in females compared to males, suggesting post-transcriptional regulation of the four circRNAs analyzed. MLLT3 showed no differences in expression, consistent with its corresponding circRNA. By contrast, for the upregulated transcripts, we observed a coherent change in expression between *hsa_circ_0140253* and the corresponding linear transcript *LOC_389906* (Fig. 3B).

Next, we checked whether the differentially expressed circRNAs were detectable in serum (Fig. 3C-D**)** and whether the levels of the circRNAs detected in PBMCs may be reflected by serum levels. To this end, total RNA was isolated from serum and circRNA levels were measured by RT-qPCR analysis. The levels of the circRNAs *hsa_circ_0140253* as well as the linear counterpart (*LOC_389906*) were increased, while *hsa_circ_0029426* decreased in serum purified from females affected by RRMS as compared to RRMS males, in line with the observation in PBMCs. In contrast, the levels of *hsa_circ_0005354* and *hsa_circ_0002082* were not influenced by sex (Fig. 3C-D) in serum samples. Interestingly, we also analyzed the linear mRNAs and found that, except for *LOC_389906*, described above, the mRNAs analyzed could be detected in serum but did not show statistically significant changes (Fig. 3C-D).

*Hsa_circ_0140253* is encoded on chromosome X, and thus the differential expression observed in male and female MS samples could be linked to the ability of the *hsa_circ_0140253*-encoding gene to escape X-chromosome inactivation or could be related to a gene-specific regulation associated with MS pathogenesis. Understanding this point is extremely relevant for a sex-biased disease such as MS, and therefore we validated the differential expression in MS samples using additional data. Initially, we selected two different cellular models isolated from male and female healthy human donors: PBMCs [10 females and 10 males (A)], and human umbilical vein endothelial cells [5 females and 5 males (B)]. Analysis of RNA levels for *hsa_circ_0140253* and *LOC_389906* in these two cell types did not show difference between males and females (Supplemental Figure S3A-B) possibly suggesting a dysregulation related to the presence of MS disease.

Next, to validate this observation in a larger dataset, we analyzed public datasets of RNA sequencing data from MS patients and HCs; the results, shown in Supplemental Figure S3C, confirmed an upregulation in female MS patients of the locus containing the gene *LOC_389906*, but not in HCs (Supplemental Figure S3C), suggesting that the upregulation of *hsa_circ_0140253* in females can be associated with the presence of MS disease in the analyzed patients.

### CircRNAs Levels and Correlation with Disease Severity

To evaluate the correlation of the validated circRNAs with MS pathology, we studied changes in the levels of the circRNAs relative to disease severity using the two different forms of MS, RRMS and SPMS, as readout. RT-qPCR analysis was performed on the cohort described in Table 1, including 4 males and 4 females diagnosed with RRMS with EDSS < 4.5, and 4 males and 4 females diagnosed with SPMS with EDSS > 4.5.

**Table 1:** Main characteristics of the individuals enrolled in the severe diseases set

Severe diseases set	RRMS edss < 4.5	SPMS edss > 4.5
No. tot	8	8
Female	4	4
Male	4	4
Female - Age, yrs, mean ± SD	40.25 ± 13.27	62 ± 4.24
Male - Age, yrs, mean ± SD	47.87 ± 15.28	69.25 ± 5.12
Female - Disease duration, mean ± SD	3.41 ± 4.83	25.75 ± 11.32
Male- Disease duration, mean ± SD	5 ± 2.54	21.25 ± 8.57
Fermale- EDSS, mean ± SD	2.2 ± 0.91	6.37 ± 0.48
Male - EDSS, mean ± SD	2.5 ± 1.22	6.37 ± 0.25

Abbreviations: *SD* standard deviation, *EDSS* Expanded Disability Status Scale, *RRMS* relapsing–remitting MS, *SPMS *secondary progressive MS

**Fig. 4:**
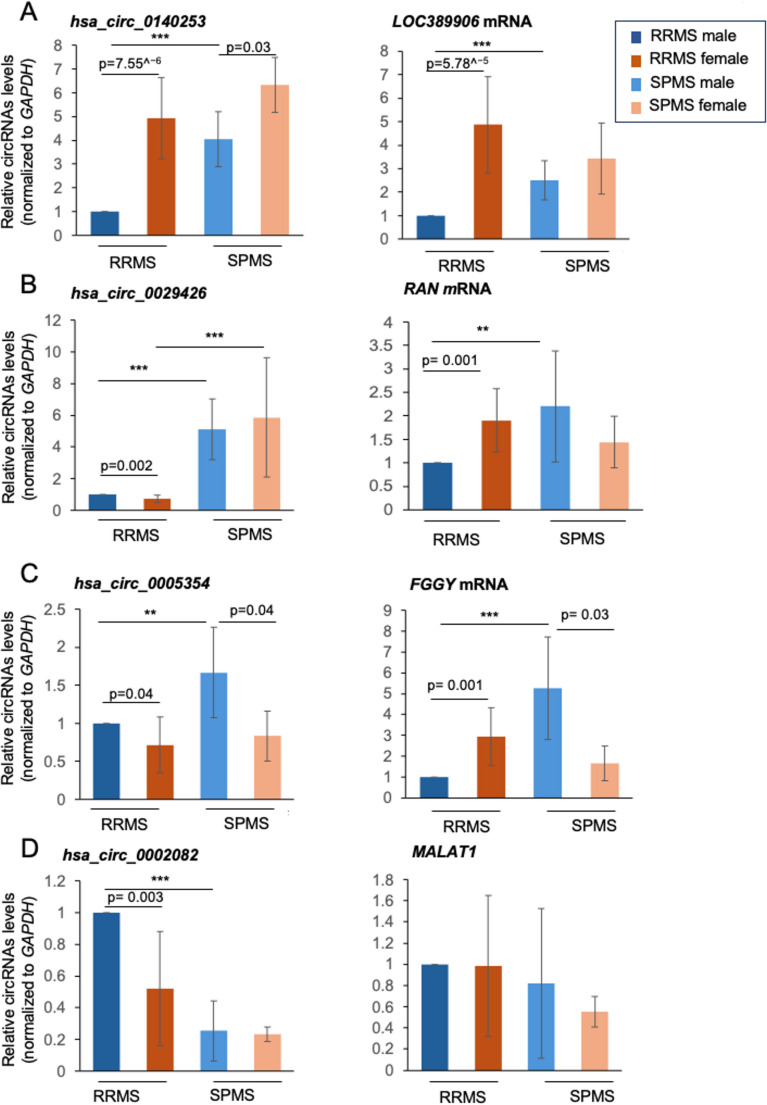
Validation of circRNA expression in PBMCs and correlation with disease severity. The levels in PBMCs of circRNAs and cognate host linear RNAs in female and male MS patients with different disease severity (RRMS and SPMS) were measured by RT-qPCR analysis. (**A**) Levels of hsa_circ_0140253 and LOC389906. (**B**) Levels of hsa_circ_0029426 and RAN mRNA. (**C**) Levels of hsa_circ_0005354 and FGGY mRNA. (**D**) Levels of hsa_circ_0002082 and MALAT1

As shown, *hsa_circ_0140253* levels remained elevated in females as compared to males, as the disease progressed to SPMS; this pattern was not observed for the linear counterpart (Fig. 4A). We also observed a consistent and significant reduction of *hsa_circ_0005354* in both RRMS and SPMS patients in females as compared to males (Fig. 4C). By contrast, *hsa_circ_0029426* and *hsa_circ_0002082* did not show significant differences when analyzing samples purified from SPMS patients (Fig. 4B and D, left panel). Additionally, among the linear transcripts, only *FGGY* showed differential expression between female and male patients in both RRMS and SPMS. Specifically, *FGGY* was upregulated in female RRMS patients and downregulated in females with SPMS compared to males. In contrast, no significant differences were observed in the expression of the linear parent transcripts, *RAN* mRNA, or *MALAT1* in SPMS patients (Fig. 4B–D, right panels).

Comparison of the two sexes as the disease progressed from RRMS to SPMS showed an increase in *hsa_circ_0140253*,* hsa_circ_0029426*,* hsa_circ_0005354*, while *hsa_circ_0002082* levels declined. In males, the linear parent transcripts showed the same trends as the circRNAs (Fig. 4A-D, right panels). In summary, these preliminary results suggest an overall change in the expression of the validated circRNAs during disease progression; however, it is important to consider that the RRMS and SPMS groups differed in age and disease duration, which may represent potential confounding factors when interpreting the results.

The levels of circRNAs and mRNAs were normalized to GAPDH mRNA levels. Data are represented as the means ± standard deviation (+SD) from 8 RRMS and 8 SPMS samples (4 males and 4 females per group). Statistical significance between groups was assessed using a two-tailed Student’s t-test (** *p* < 0.01, *** *p* < 0.001). Abbreviations: RRMS, relapsing–remitting MS; SPMS, secondary progressive MS.

Given that the impact of sex on the effectiveness of DMTs has been largely overlooked, we performed a preliminary investigation to explore how sex influences circRNA expression levels in males and females treated with different DMTs or left untreated. RNA samples purified from PBMCs of patients affected by RRMS and undergoing treatment with different DMTs (DMF, FTY, IFN, NAT, and TER) or UNT were assessed by RT-qPCR analysis (Supplemental Table 2).

**Fig. 5:**
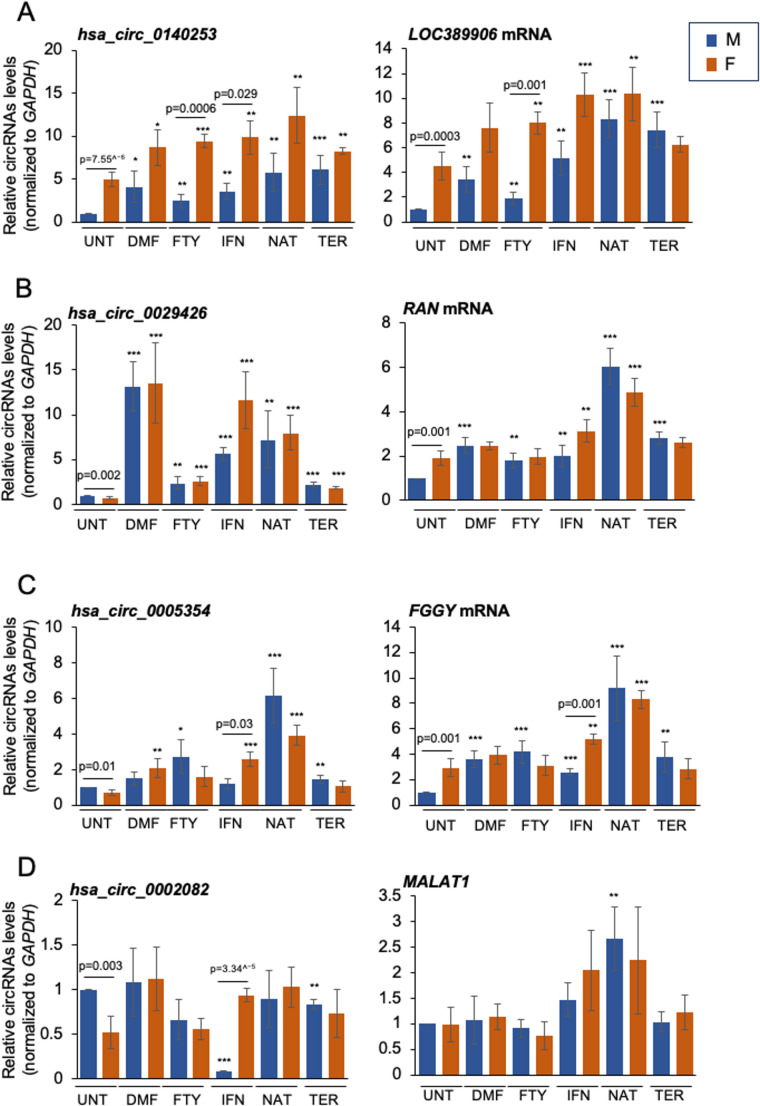
Validation of circRNA expression and cognate host linear RNAs in PBMCs and correlation with different DMTs. The levels in PBMCs of circRNAs and cognate host linear RNAs in female and male MS patients treated with different DMTs were measured by RT-qPCR analysis. (**A**) Levels of hsa_circ_0140253 and LOC_389906. (**B**) Levels of hsa_circ_0029426 and RAN mRNA. (**C**) Levels of hsa_circ_0005354 and FGGY mRNA. (**D**) Levels of hsa_circ_0002082 and MALAT1. The levels of circRNAs and linear RNAs were normalized to GAPDH mRNA levels. Data are represented as the means ± standard deviation (+SD) from 8 RRMS and 8 SPMS samples (4 males and 4 females per group). Statistical significance between groups was assessed using a two-tailed Student’s t-test ( * *p* < 0.05, ** *p* < 0.01, *** *p* < 0.001) represent UNT male versus male treated with different DMTs and UNT female versus female treated with different DMTs. Abbreviations: UNT, untreated; DMF, dimethyl fumarate; FTY, fingolimod; IFN, interferon; NAT, natalizumab; TER, teriflunomide; RRMS, relapsing–remitting MS; SPMS, secondary progressive MS

We found that *hsa_circ_0140253* was differentially expressed in UNT and patients treated with FTY (*p* = 0.0006) and IFN (*p* = 0.029); and its linear counterpart, *LOC_389906* showed a similar trend (Fig. 5A). *Hsa_circ_0029426* and *RAN* mRNA did not show differences when analyzing different therapeutic regimens (Fig. 5B). Interestingly, for *hsa_circ_0005354* and *hsa_circ_0002082* IFN appears to reverse the expression patterns, as these circRNAs were lower in females relative to males in UNT, but became significantly higher in females relative to males after IFN treatment (Fig. 5C and D). The linear counterparts showed significant differences only when analyzing *FGGY* mRNA in patients treated with IFN (Fig. 5C), while *MALAT1* remained generally unchanged (Fig. 5D). Moreover, *hsa_circ_0140253* increased significantly in female MS patients treated with different DMTs compared to UNT females; the same trend was observed for males (Fig. 5A). We observed similar trends for *hsa_circ_0029426* and *hsa_circ_0005354* in both sexes (Fig. 5B-C). In contrast, *hsa_circ_0002082* was downregulated in male MS patients treated with IFN and TER compared to UNT male samples (Fig. 5D). The linear transcripts exhibited the same expression trend as their corresponding circRNAs, except for *MALAT1*.

### Definition of a circRNA-miRNA-mRNA Network

**Fig. 6:**
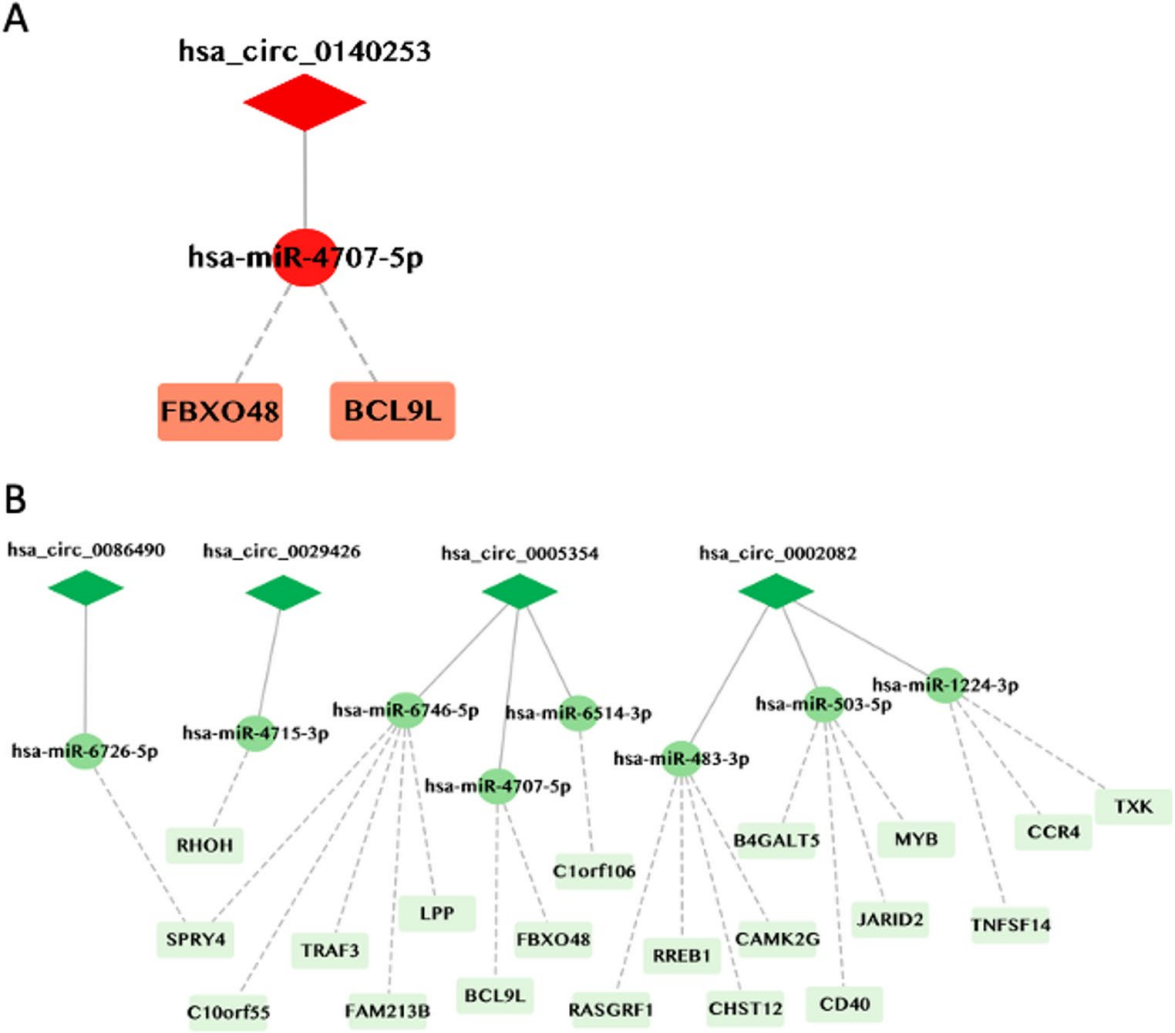
Networks of circRNA-miRNA-mRNA affecting MS-associated proteins. (**A**) Network of elevated circRNAs and (**B**) reduced circRNAs. CircRNAs are represented as red or green diamonds, miRNAs as red or green circles, and mRNAs as light red or light green rectangles. Red represents networks generated from upregulated circRNAs and green from downregulated circRNAs

Highly expressed circRNAs have been shown to interact with miRNAs and RBPs, thus regulating gene expression (Ikeda et al. 2023). Therefore, we identified miRNAs and RBPs interacting with these circRNAs using the prediction program miRanda and the web tool CircInteractome, respectively (Dudekula et al. 2016) as described in Lodde et al., (Lodde et al. 2024) (Supplemental Figure S4). We then studied possible links between these circRNAs, the predicted miRNA binding to them, and potential mRNAs affected in turn. We focused on putative mRNA targets identified by the International Multiple Sclerosis Genetics Consortium (International Multiple Sclerosis Genetics Consortium 2019) and constructed a putative circRNA-miRNA-mRNA network underlying dysregulated pathways in MS patients. As shown in Fig. 6, in silico networks generated from the upregulated circRNA *hsa_circ_0140253* are predicted to alter the expression levels of *FBXO48* and *BCL9L* mRNAs through its predicted actions on miR-4707-5p. Interestingly, the encoded protein FBXO48 is involved in SCF-dependent proteasomal ubiquitin-dependent protein degradation, while BCL9L is associated with B cell function, both proteins previously associated to MS (Fig. 6A) (Klose et al. 2023; Patsopoulos et al. 2011). The in silico networks were generated by analyzing the identified circRNAs downregulated in females as compared to males point to sequestration of miR-6726-5p, -4715-3p, -6746-5p, -4707-5p, -6514-3p, -483-3p, -503-5p and - 1224-3p, which in turn are predicted to modulate the expression of multiple MS proteins including CD40, TNFSF14 and TRAF3, which have key functions in the regulation of the immune system (Fig. 6B). The differential regulation of the putative circRNA targets supports a sex-specific pattern of MS. While competing endogenous RNA (ceRNA) analyses provide valuable hypotheses, their interpretation requires caution, as these networks are entirely based on in silico predictions and should be regarded as hypothesis-generating. They lack experimental validation and integration with matched miRNA/mRNA expression data, and do not account for intracellular stoichiometry or the spatial availability of regulatory RNAs. Nevertheless, these predicted networks offer a compelling framework for understanding how sex-biased circRNAs may modulate immune-related pathways in MS. Further experimental validation is needed to assess their biological relevance and to determine whether these candidate interactions can be reproduced in independent, larger cohorts.

## Discussion

To explore whether sex influences circRNA levels in newly diagnosed RRMS patients, in this pilot study we performed a sex-stratified analysis of previously published circRNA microarray data (Lodde et al. 2024) and identified circRNAs differentially expressed in female MS patients as compared to male MS patients. Specifically, we found 33 circRNAs differentially expressed, 6 upregulated and 27 downregulated in female patients as compared to male patients. Validation of these results in an independent cohort comprising 8 female RRMS and 8 male RRMS patients confirmed that *hsa_circ_0140253* levels were increased, whereas the levels of *hsa_circ_0029426*, *hsa_circ_0005354*, and *hsa_circ_0002082* were decreased in females as compared to males. Interestingly, the differential expression (females vs. males) was observed only in MS patients and not in HCs. The expression of circRNAs was also analyzed in serum, where we found that two validated circRNAs (*hsa_circ_0140253* and *hsa_circ_0029426*) were also differentially expressed in male as compared to female MS patients in serum. Notably, *hsa_circ_0140253* was confirmed to be upregulated in female MS patients after all the validation steps, even when the disease course changes from RRMS to SPMS. Investigation of the impact of sex on circRNA levels in male and female MS patients treated with different DMTs or untreated, revealed the most notable sex differences in patients receiving IFN treatment, where *hsa_circ_0140253*,* hsa_circ_0005353*,* and hsa_circ_0002082* were significantly elevated in females compared to males. Interestingly, *hsa_circ_0140253* showed higher expression in females across all treatments, with significant upregulation specifically in the UNT group, as well as in those patients receiving FTY and IFN treatments. Unfortunately, due to the limited sample size, this pilot study provides an initial insight into potential sex-related differences in circRNA expression in MS, which may provide a useful basis to guide future validation efforts in larger and more stratified cohorts. Moreover, limited information is currently available in MS regarding *hsa_circ_0140253*, and thus we do not know the impact of this pattern of expression. Rybak-Wolf et al. identified the *hsa_circ_0140253* as being highly expressed in the mammalian brain (Rybak-Wolf et al. 2014). Despite its high expression, the function and specific expression patterns of *hsa_circ_0140253* within the nervous system remain to be elucidated. *Hsa_circ_0140253* is transcribed from the *LOC389906* gene, which corresponds to the Zinc Finger Protein 839 Pseudogene located on the X chromosome and was described by Katsir et al. as a potential gene escaper from X-inactivation (Wainer Katsir and Linial 2019). The differential expression of *hsa_circ_0140253* can thus be related to gene expression regulation specific to female MS or MS modulation of escape. Due to the relevance of X-chromosome dysregulation in autoimmunity, we studied *hsa_circ_0140253* and the *LOC389906* transcripts in PBMCs and HUVECs derived from both healthy male and female donors. Furthermore, using public RNAseq datasets, we observed an upregulation in the *hsa_circ_0140253* and *LOC389906* RNA expression in females compared to males in samples from MS patients but not from HCs (Supplemental Figure S3). *Hsa_circ_0140253* was the only significantly dysregulated circRNA identified on the X chromosome in our dataset, and it deserves mention due to its genomic location, as increasing evidence suggests that the higher prevalence of autoimmune diseases in females compared to males may be linked to the presence of the X chromosome (Fairweather et al. 2024). Moreover, several immune-related genes encoded on the X chromosome have been associated with a heightened likelihood of autoimmune disease in females (Greer and McCombe 2011). Further validation of *hsa_circ_0140253* and the *LOC389906* gene is essential to better understand their potential roles and functions in the pathogenesis of MS.

The validated circRNAs downregulated in female MS patients are transcribed from different host genes — *RAN*, *FGGY* and *MALAT1*. *Hsa_circ_0029426* is transcribed from the gene encoding RAN, a member of the RAS oncogene family. RAN is a small GTPase that facilitates nucleocytoplasmic transport and mitotic spindle assembly (Patsopoulos et al. 2011). Mislocalization and/or aggregation of nucleocytoplasmic transport proteins have been observed in multiple models of amyotrophic lateral sclerosis (ALS) and Alzheimer’s disease (AD), both in cultured cells and in live organisms (Sheng et al. 2019; Vanneste and Van Den Bosch 2021). Mastroeni and colleagues found a significant impairment in nuclear transport of DNMT1 and RNA polymerase II within neurons vulnerable to AD pathology (Mastroeni et al. 2013). *Hsa_circ_0029426*, which is transcribed from the *RAN* gene, was less expressed in both PBMCs and serum purified from female MS patients compared to males, but it was elevated as the disease progressed. *Hsa_circ_0029426* has been studied in various cancers, particularly in glioblastoma, where its expression was found to be closely associated with clinical severity and prognosis (Zhang et al. 2019). Functionally, *hsa_circ_0029426* has been shown to significantly enhance cell proliferation, migration, and invasion, while suppressing cell apoptosis (Wang et al. 2018; Zhang et al. 2019).

Besides hosting *hsa_circ_0005354*, the *FGGY* gene encodes the Carbohydrate Kinase Domain-Containing Protein, primarily studied in the context of ALS (Van Es et al. 2009). In a GWAS meta-analysis of blood neurofilament light chain (NfL) levels from eleven different cohorts, Shahzad Ahmad et al. identified a locus within the FGGY gene that had been previously associated with sporadic ALS (Ahmad et al. 2024). Blood levels of NfL have gained recognition as a robust biomarker of neuro-axonal injury, showing increased levels across several neurodegenerative diseases, including AD, Parkinson’s disease (PD), Huntington’s disease, ALS, and MS. *Hsa_circ_0005354* levels were lower in PBMCs of female MS patients compared to male MS patients and remained low in females with MS who had RRMS or the more advanced form, SPMS. *Hsa_circ_0005354* was found to be reduced in pediatric acute myeloid leukemia (AML) patients and is associated with an increased risk of developing AML (Ye et al. 2023). However, no other studies have explored its specific role or function in detail.

*The hsa_circ_0002082*, also known as *circ-MALAT1*, was lower in PBMCs purified from MS females as compared to males, and is generated from the long noncoding (lnc)RNA *MALAT1* (Metastasis-Associated Lung Adenocarcinoma Transcript 1). *Hsa_circ_0002082* has been studied in various cancers. Yu Liu et al. demonstrated that loss of *hsa_circ_0002082* inhibits breast cancer growth and cell proliferation, induces apoptosis and migration/invasion changes, as well as epithelial-mesenchymal transition in culture (Liu et al. 2022). Chen et al. reported elevated expression of *hsa_circ_0002082* in hepatocellular carcinoma (HCC) cancer stem cells (CSCs), where it enhances CSCs self-renewal at least in part by activating the JAK2/STAT3 signaling pathway (Chen et al. 2020). The linear lncRNA *MALAT1* has been studied for its potential role in the development of various autoimmune diseases. *MALAT1* has a role in a wide range of cellular functions, including cell proliferation, differentiation, apoptosis, and inflammation (Mohan et al. 2024). Specific genetic variants of *MALAT1* have been associated with an increased risk of MS (Eftekharian et al. 2019). Fenoglio et al. observed extensive dysregulation of multiple lncRNAs in MS patients relative to controls; in particular, *MALAT1*, *MEG9*, *NRON*, *ANRIL*, *TUG1*, *XIST*, *SOX2OT*, *GOMAFU*, *HULC*, and *BACE-1AS* were significantly downregulated in MS patients (Fenoglio et al. 2018). In our data, we observed a trend toward downregulation of *MALAT1* either in PBMCs and serum of female MS patients compared to male MS patients, though this difference was not statistically significant. Our data suggest that the differential expression of *MALAT1* is influenced primarily by the presence of MS rather than by sex.

We further investigated whether different MS treatments impact the expression levels of the circRNAs of interest by analyzing their levels in PBMCs of MS patients, female and male patients undergoing different DMT drugs. Each of the DMT drugs for MS treatment appears to have a different functional profile, and their efficacy depending on sex has been largely overlooked. Our preliminary data can be considered only exploratory due to the limited size of the cohort analyzed for each treatment subgroup. *Hsa_circ_0140253* was differentially expressed in UNT relative to patients treated with FTY and IFN, while *hsa_circ_0005354* and *hsa_circ_0002082* showed that IFN seems to reverse circRNA expression patterns, as both circRNAs were lower in females as compared to males in UNT. Based on these preliminary observations, it may be important to consider patient drug treatment, as DMTs could potentially affect sex-associated circRNA expression levels. Therefore, further analyses in larger and more stratified cohorts will be necessary to better clarify these associations. Several studies have demonstrated that ncRNAs, including circRNAs and miRNAs, may play a role in various stages of MS development. CircRNAs, in particular, can interact with miRNAs, affecting the levels of their mRNA targets and contributing to the formation of ceRNA networks. In this context, we performed an in silico prediction of the circRNA-miRNA-mRNA network for MS risk-associated genes identified by the International Multiple Sclerosis Genetics Consortium (International Multiple Sclerosis Genetics Consortium 2019). This analysis is entirely based on computational predictions and should therefore be interpreted as preliminary especially with regard to the intracellular stoichiometry of regulatory RNAs. The potential role of certain MS-related circRNAs in regulating MS pathogenesis through possible interaction with miRNAs warrants further investigation. *Hsa_circ_0140253* is predicted to result in altered expression of FBXO48 and BCL9L through possible sequestration of miR-4707-5p. FBXO48 (F-Box Protein 48) is involved in the ubiquitin–proteasome protein-degradation pathway, which has been implicated in various neurodegenerative diseases (Ilyin et al. 2000). BCL9L (B cell CLL/lymphoma 9-like) is associated with B cell functions, a key cell type involved in MS pathogenesis (Steri et al. 2017), and acts as a transcriptional coactivator of β-catenin, playing an essential role in the Wnt/β-catenin signaling pathway. BCL9L expression is elevated in various human cancers, and inhibiting BCL9/BCL9L can enhance antitumor responses (He et al. 2024). The networks generated by analyzing the downregulated circRNAs in females as compared to males point to a possible sequestration of miR-6726-5p, -4715-3p, -6746-5p, -4707-5p, -6514-3p, -483-3p, -503-5p and - 1224-3p, which in turn are predicted to modulate the expression of multiple MS-associated proteins, including TNFSF14, CXCR4 and TRAF3 which have key functions in the regulation of the immune system. TNFSF14, a newly identified risk gene for MS (Zuccalà et al. 2021), plays a crucial role in the survival of CD4⁺ memory T cells, a cell population known to be important in MS pathogenesis (Chitnis 2007). CXCR4 in CNS can be regulated by modulating SDF1α at the blood–brain barrier. In active MS lesions, the CXCR4/SDF1α axis has been implicated in facilitating the infiltration of pathogenic cells into the CNS (Galli et al. 2019). TRAF3, a key regulator in immune-related signal transduction, interacts with various regulatory proteins, kinases, and receptors to elicit its effects (Häcker et al. 2011). However much more work is needed to establish whether *hsa_circ_0140253* or other MS-associated circRNAs interact with miRNAs and whether such predicted interactions have biological relevance in MS pathogenesis. Additional experimental studies are necessary to assess the biological significance of these networks and to verify whether the proposed interactions can be consistently reproduced in larger, independent cohorts.

In closing, in this pilot study we have examined circRNA expression levels with a specific focus on sex differences in newly diagnosed MS patients. To date, the only other study addressing circRNA expression differences, conducted by Iparraguirre and colleagues in 2020, compared female MS patients with female HCs and male MS patients with male HCs, but it did not address sex-based differences within the MS patient group (Iparraguirre et al. 2020). Both that study and ours share one important limitation: the small size of the discovery dataset and subtle unaccounted variability between individuals may also contribute to the observed expression patterns. However, such potential sources of heterogeneity are difficult to fully address in small cohorts. Indeed, our initial analysis included only 3 female MS samples and 2 male MS samples, with a subsequent validation conducted on a larger cohort of 8 female and 8 male MS samples. While the present work offers initial evidence of sex-driven circRNA expression at the time of MS diagnosis, additional investigations, with larger and well-characterized cohorts, are necessary to further characterize the expression profiles, determine the biological relevance of these patterns and to clarify the molecular pathways involved in MS pathogenesis. This study has several limitations that should be acknowledged. The relatively small sample size, particularly within treatment-specific subgroups, limits statistical power and generalizability and may introduce variability related to individual heterogeneity. In addition, differences in age and disease duration between RRMS and SPMS groups may represent potential confounding factors, and the limited number of patients per DMT subgroups precludes definitive conclusions regarding treatment-specific sex effects. Nevertheless, the study is strengthened by the use of an independent validation cohort, the integration of both PBMC and serum analyses, and a sex-stratified study design, which together provide a robust exploratory framework for future larger and well-characterized investigations.

In summary, this exploratory study provides preliminary evidence of sex-related differences in circRNA expression at MS onset and their possible association with disease severity and exposure to different DMTs. However, these findings should be interpreted with caution, and further in-depth analyses and functional studies are required to better clarify their biological relevance in MS. Overall, the results suggest a link between circRNA expression and sex differences in MS, underscoring the importance of sex-stratified cohorts in future studies of disease pathogenesis.

## Supplementary Information

Below is the link to the electronic supplementary material.

Supplementary Material 1

## Data Availability

All data needed to evaluate the conclusions in the paper are present in the paper and in the Supplementary Materials.
