# Supplementary material for: Sex-Related Differences in Circular RNA Expression in Multiple Sclerosis: A Pilot Study
Source: J Mol Neurosci. 2026 May 26;76(2):90. doi: 10.1007/s12031-026-02548-3 (PMC13212822; doi:10.1007/s12031-026-02548-3)
Supplement: Supplementary file 1 — Supplementary Material 1 [file 12031_2026_2548_MOESM1_ESM.docx]

**Supplemental Figures**

**Sex-Related Differences in Circular RNA Expression in Multiple Sclerosis: a pilot study**

Valeria Lodde ^1,^*, Igrazio Roberto Zarbo ^2,3^, Gabriele Farina ^3^, Enrico Zoroddu^1^, Paolo Solla ^2,3^, Giuseppe Delogu ^1^, Myriam Gorospe ^4^, Matteo Floris ^1^, Ilaria Campesi ^1^, Maria Laura Idda ^1^

^1^ Department of Biomedical Sciences, University of Sassari, Sassari 07100, Italy

^2^ Department of Medicine, Surgery and Pharmacy, University of Sassari, Sassari 07100, Italy

^3^ Unit of Clinical Neurology, AOU Sassari, Sassari 07100, Italy

^4^ Laboratory of Genetics and Genomics, National Institute on Aging Intramural Research Program, National Institutes of Health, Baltimore, MD 21224, United States

***** Correspondence author: Valeria Lodde: [vlodde@uniss.it](mailto:vlodde@uniss.it)

**Supplemental Figure S1**: Overview of the workflow and different cohorts used in the study.

**Supplemental Figure S2:** Table showing the list of circRNAs differentially abundant in Female HCs compared to male HCs, depicting the top 5 elevated and 7 reduced circRNAs.

**Supplemental Figure S3:** hsa_circ_0140253 and LOC_389906 levels in healthy PBMCs (10 females and 10 males) **(A)**, and healthy HUVEC **(B)** (5 females and 5 males). **(C)** expression of LOC_389906 analyzed in public RNAseq dataset from PBMCs of female MS patients compare to male MS patients and in female HCs compared male HCs.

**Supplemental Figure S4:** Identification of the miRNAs and RBP Targets. (A) Schematic representation of circRNAs with putative miRNA binding site (MRE) and RNA-binding protein binding site (RBP-bs). (B,C) Tables showing lists of human circRNAs identified from our studies and target miRNAs and interacting RNA-binding proteins, as determined by analysis performed using miRanda and circInteractome, respectively.

**Supplemental Table 1**: RNA-specific primer pairs.

| RT-(q)PCR primers | Primer Sequences (5’ - 3’) |
| --- | --- |
| hsa_circ_0140253 | FW: GCTTACCTGGAATCACGTGTC |
|  | RV: GGCCATTCCTGACCTTTTGTT |
| hsa_circ_0086490 | FW: TGCTTCTTCACCCCTACATCA |
|  | RV: TGGTCTGGGATGGTGTGAAG |
| hsa_circ_0029426 | FW: GTGAAACGTCATTTGACTGGT |
|  | RV: TTTCCAGTACCACCATCACCA |
| hsa_circ_0005354 | FW: TCTCCCGGACTTCTTATCGTG |
|  | RV: GTCCACACCCACATAGTACCT |
| hsa_circ_0002082 | FW: GCTGAGTGATAAAGGCTGAGTG |
|  | RV: TTCGTTCTTCCGCTCAAATCC |
| LOC_389906 | FW: CCTGCCATTCAACAAACGCATT |
|  | RV: GACTACTGAGGGCATCTGGAA |
| MLLT3 | FW: ACGACTGGATGGTGTTCGTAC |
|  | RV: GCTTTCGTGCAAGTGGAAGA |
| RAN | FW: GGTTCATCCCCTAGTGTTCCA |
|  | RV: CCATCTCTCAGTCCACCGAAT |
| FGGY | FW: TACTATGTGGGTGTGGACGTT |
|  | RV: CTGCTCATGGTGGTTGAACTG |
| MALAT1 | FW: TGGGGGAGTTTCGTACTGAG |
|  | RV: TCTCCAGGACTTGGCAGTCT |
| GAPDH | FW: ATTTGGTCGTATTGGGCGCC |
|  | RV: TTGAGGTCAATGAAGGGGTC |

**Supplemental Table 2. Main characteristics of the individuals enrolled in the therapy set.**

| **Therapy set** | **UNT** | **DMF** | **FTY** | **IFN** | **NAT** | **TER** |
| --- | --- | --- | --- | --- | --- | --- |
|  |  |  |  |  |  |  |
| **No. tot** | 8 | 8 | 8 | 8 | 8 | 8 |
| **Female** | 4 | 4 | 4 | 4 | 4 | 4 |
| **Male** | 4 | 4 | 4 | 4 | 4 | 4 |
| **Female - Age, yrs, mean ± SD** | 40.25 ± 13.27 | 54 ± 3.16 | 45.25 ± 7.27 | 47 ± 11.28 | 31.75 ± 6.70 | 50 ± 4.24 |
| **Male - Age, yrs, mean ± SD** | 47.87 ± 15.28 | 46.25 ± 8.46 | 46.25 ± 11.61 | 44 ± 10.51 | 37.75 ± 8.18 | 56 ± 6.97 |
| **Female - Disease duration, mean ± SD** | 3.41 ± 4.83 | 6 ± 1.82 | 16.25 ± 3.77 | 13.75 ± 8.95 | 10.5 ± 4.04 | 14.75 ± 2.98 |
| **Male - Disease duration, mean ± SD** | 5 ± 2.54 | 9 ± 2.94 | 12.25 ± 4.92 | 11 ± 4.83 | 11.25 ± 3.86 | 8.25 ± 4.34 |
| **Fermale- EDSS, mean ± SD** | 2.2 ± 0.91 | 1.5 ± 0.40 | 1.65 ± 0.47 | 1.62 ± 0.75 | 1.75 ± 0.64 | 2.37 ± 0.75 |
| **Male - EDSS, mean ± SD** | 2.5 ± 1.22 | 1.62 ± 0.47 | 2.5 ± 1.29 | 2.37 ± 0.75 | 1.87 ± 0.62 | 2 ± 0.81 |

Abbreviations: UNT, untreated; DMF, dimethyl fumarate; FTY, fingolimod; IFN, interferon; NAT, natalizumab; TER, teriflunomide; SD, standard deviation; EDSS, Expanded Disability Status Scale.
